# Focal Myositis of the Masseter Muscle Masquerading as Temporomandibular Disorder: Diagnostic Challenges and Management

**DOI:** 10.7759/cureus.88768

**Published:** 2025-07-25

**Authors:** Shinya Matsuda, Masayuki Motohashi, Ayaka Ishii, Hideaki Sato, Makoto Adachi

**Affiliations:** 1 Oral and Maxillofacial Surgery, Asahikawa Medical University, Asahikawa, JPN; 2 Oral and Maxillofacial Surgery, Asahikawa City Hospital, Asahikawa, JPN; 3 Oral and Maxillofacial Surgery, Nagoya Tokushukai General Hospital, Kasugai, JPN

**Keywords:** focal myositis, head and neck, masseter muscle, temporomandibular disorder, trismus

## Abstract

Focal myositis (FM) is a rare, localized inflammatory muscle disease that is often incorrectly diagnosed due to nonspecific symptoms, especially in the head and neck region. We present a case of FM involving the masseter muscle in a 76-year-old man, which was initially presumed to be a temporomandibular joint disorder. The patient exhibited trismus and swelling of the right cheek. Magnetic resonance imaging (MRI) showed masseter muscle swelling and high signal intensity on diffusion-weighted image (DWI). Histopathological findings showed FM with moderate inflammatory cell infiltration and muscle atrophy. Empirical treatment with sulbactam sodium and ampicillin sodium was administered, followed by corticosteroid treatment with prednisolone, which significantly improved the symptoms. Persistent trismus remained due to fibrosis, highlighting the importance of early diagnosis and intervention. This case highlights the importance of considering FM in the differential diagnosis of unilateral masticatory muscle swelling and trismus. Imaging and histopathological findings are necessary for accurate diagnosis, and early corticosteroid therapy is essential to prevent sequelae such as fibrosis. A multidisciplinary approach is essential for effective management.

## Introduction

Focal myositis (FM) is a rare, localized inflammatory muscle disorder first described by Heffner et al. in 1977 [1]. The etiology of FM is unknown. Unlike systemic myopathies, FM affects a single muscle or muscle group but does not affect the entire body. The disease is characterized by lymphocytic infiltration, muscle fiber necrosis, and fibrosis [2]. FM most commonly affects the lower extremities, but the head and neck is rare, accounting for less than 10% of cases [3,4].

The clinical presentation of FM often mimics more common conditions such as neoplastic lesions of vascular or inflammatory origin and soft-tissue sarcoma, due to overlapping symptoms such as localized swelling, pain, and impaired function [5]. Particularly in the head and neck region, FM has similar clinical symptoms such as trismus and swelling, which closely resemble temporomandibular disorders (TMD), leading to early incorrect diagnosis and delayed treatment [6]. This diagnostic challenge is further complicated by the rarity and nonspecific findings of FM, which can lead to delayed diagnosis. Although magnetic resonance imaging (MRI) plays an important role in identifying edema and fibrosis, histopathological diagnosis remains the gold standard for confirmation [2]. Histopathological examination is essential for a definitive diagnosis of FM, as it allows differentiation from other inflammatory or neoplastic diseases through the identification of characteristic inflammatory infiltrates, muscle fiber necrosis, and fibrosis [7].

In this report, we describe a case of FM arising in the masseter muscle that was initially thought to be TMD, in which the diagnostic process and treatment outcome are reviewed, highlighting the unique challenges of managing FM in the head and neck region.

## Case presentation

A 76-year-old man was referred to the Department of Oral and Maxillofacial Surgery, Asahikawa Medical University, Asahikawa, Japan, for trismus while undergoing treatment for TMD at a local dental clinic. The patient’s medical history included ulcerative colitis and hepatitis B virus carrier status, with no notable family history. The patient had experienced jaw pain and clicking sounds on movement for several years. The patient had experienced jaw pain and clicking noises on movement for several years and had attempted manual manipulation of the temporomandibular joint at a local dental clinic without improvement.

**Figure 1:**
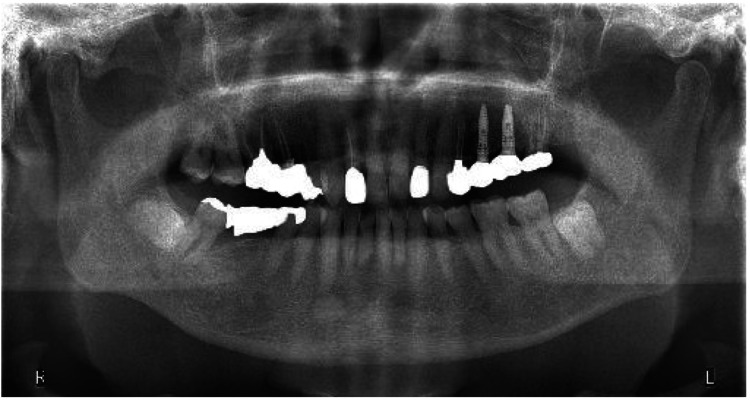
Initial panoramic radiograph.

At the first visit to our department, the patient had trismus with a maximum mouth opening of 12 mm. Clinical examination revealed no spontaneous pain, but the patient reported discomfort during mouth opening. No joint sounds were detected, although clicking was previously reported at the local dental clinic. Panoramic radiography showed no significant findings (Figure 1).

**Figure 2:**
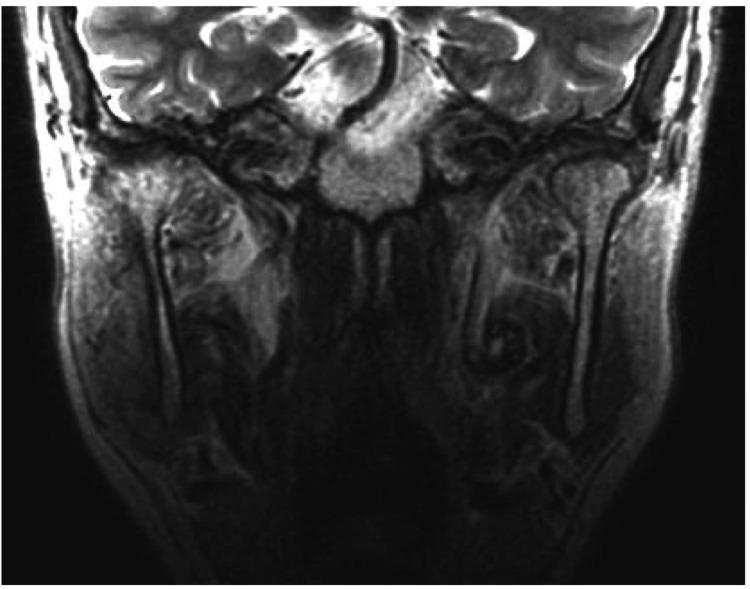
Initial MRI coronal view showing roughening and fibrosis around the right condylar process.

MRI demonstrated mild deformation of the right temporomandibular joint disc and fibrosis with roughening around the right mandibular condyle (Figure 2).

Additionally, the MRI revealed subtle edema-like changes in the right masseter muscle, which was initially considered a secondary finding. Based on these findings and the patient's history of jaw pain, TMD was diagnosed. Conservative treatment involving mouth-opening exercises was subsequently initiated, with monthly follow-up appointments. Despite consistent exercises, no improvement in mouth opening was observed during this period. Follow-up MRI examinations showed persistent edema-like changes at the periphery of the masseter muscle and surrounding tissues, with signs of fibrosis and chronic inflammation, but without significant progression.

Fifteen months later, the patient returned with worsening trismus and significant swelling of the right cheek. The patient had spontaneous pain in the right masseter region, diffuse swelling with warmth, and trismus with a maximum mouth opening of 5 mm. Body temperature was 36.1°C. Intraoral examination revealed swelling extending from around the right lower second molar to the anterior border of the ramus. Due to severe trismus, detailed examination of the right mandibular second molar was difficult; however, no tenderness on percussion, purulent discharge, or lower lip paresthesia was noted. It is worth noting that the patient's medical records from three months prior indicated a small amount of purulent discharge from the area of the right mandibular second molar, but no acute symptoms were present at that time.

**Figure 3:**
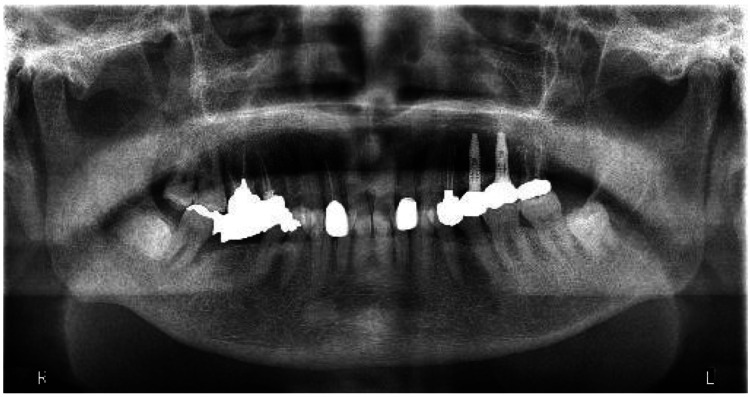
Panoramic radiograph taken 15 months after the initial visit.

Panoramic radiography revealed sclerotic changes in the right mandibular second molar region (Figure 3).

**Figure 4:**
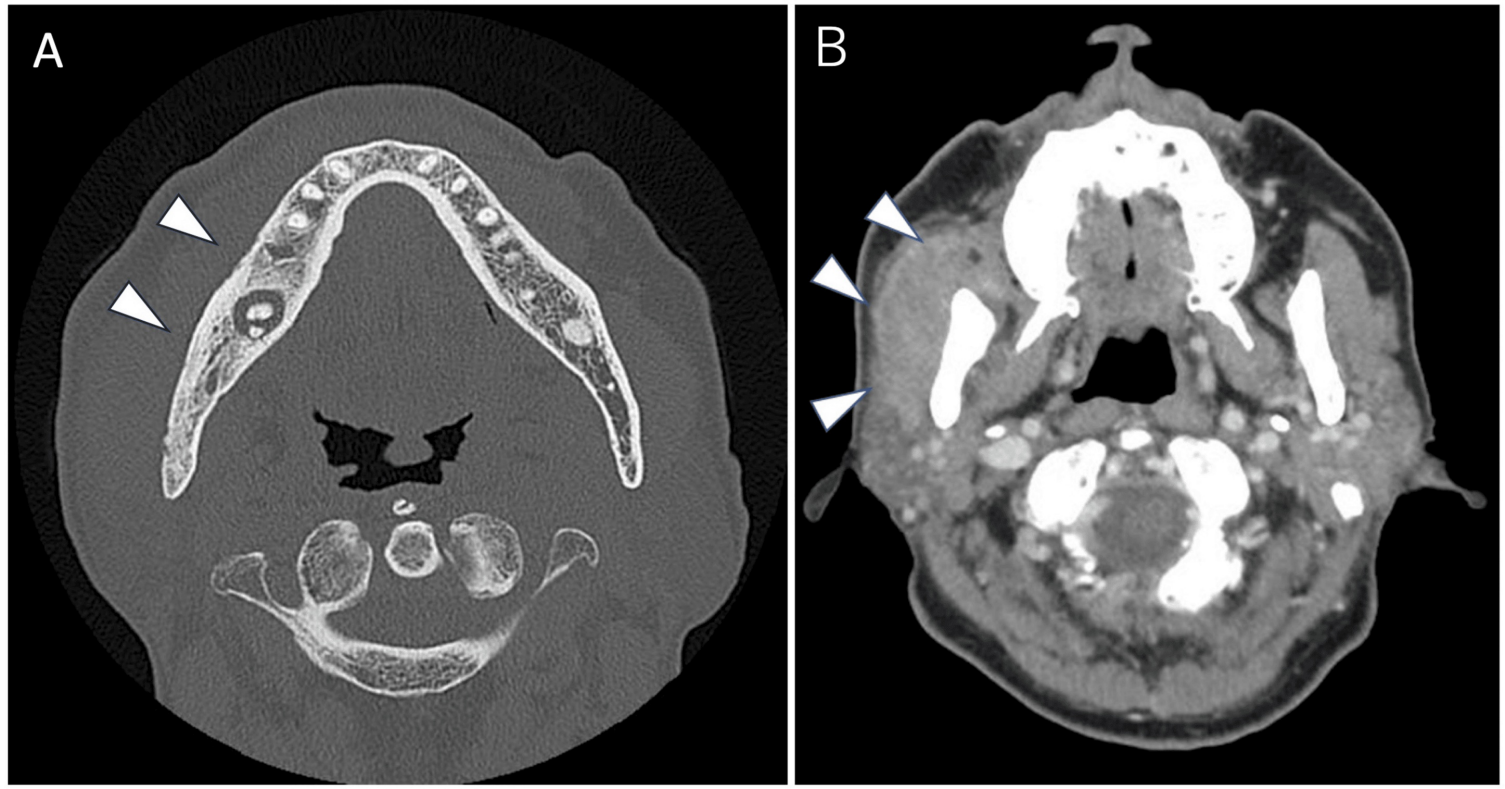
Contrast-enhanced computed tomography images of the right masseter muscle and mandible. (A) Bone sclerosis in the right mandibular second molar region (arrow). (B) Swelling of the right masseter muscle (arrow)

Contrast-enhanced computed tomography (CT) confirmed bone sclerosis around the right mandibular second molar region and showed pronounced swelling of the right masseter muscle (Figures 4A, 4B).

**Figure 5:**
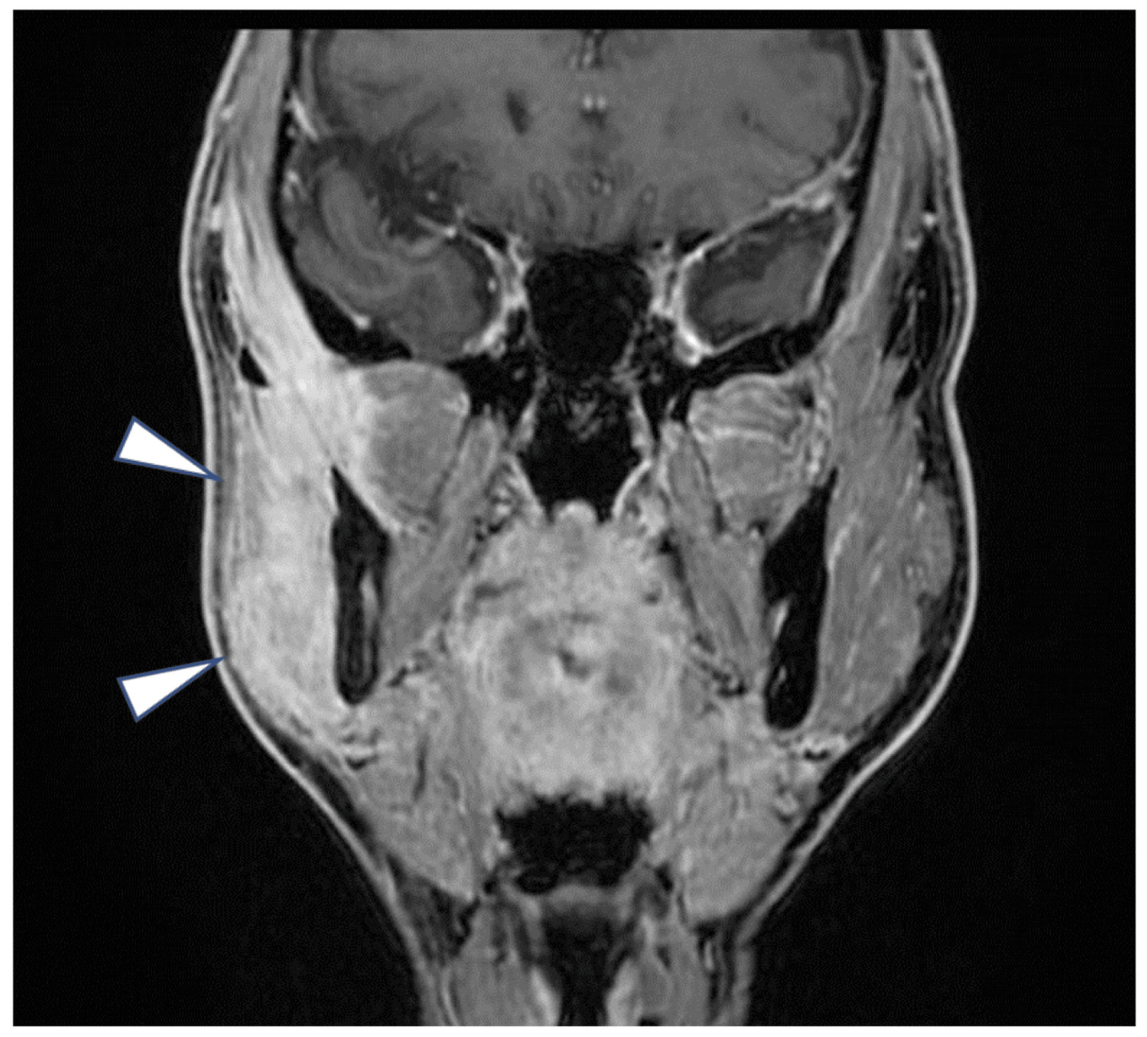
Contrast-enhanced MRI findings of the right masseter muscle. Diffusion-weighted imaging (DWI) demonstrates high signal intensity in the right masseter muscle, indicating inflammation (arrow).

Contrast-enhanced MRI showed swelling of the right masseter muscle and a high signal intensity on diffusion-weighted image (DWI) (Figure 5).

**Figure 6:**
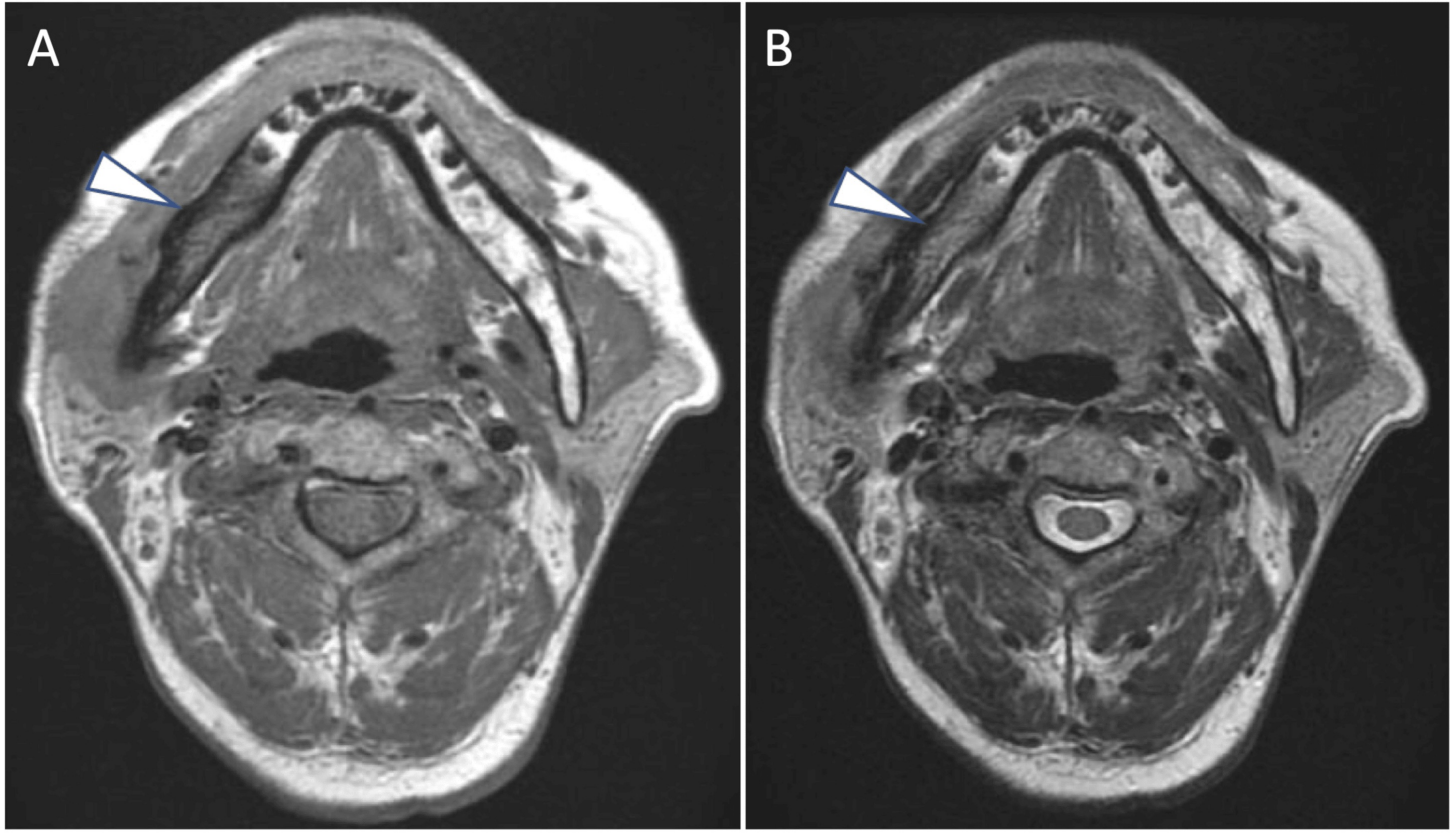
Contrast-enhanced MRI findings of the mandible. (A) T2-weighted image showing low signal intensity in the right mandibular bone, consistent with sclerosis (arrow). (B) T1-weighted image also shows low signal intensity in the mandibular bone, further supporting the diagnosis of osteomyelitis (arrow).

Additionally, the right mandible exhibited a low signal intensity on both T2-weighted and T1-weighted images (Figures 6A, 6B).

Blood tests revealed slightly elevated eosinophil counts but otherwise normal inflammatory markers and muscle enzymes. Laboratory results are summarized in Table 1.

**Table 1:** Laboratory results. WBC: white blood cell count; CRP: C-reactive protein; CK: creatine kinase; Jo-1: histidyl-tRNA synthetase

Parameter	Result	Reference Range	Unit
White blood cell count (WBC)	6,000	4,000-10,000	/μL
Neutrophil count	3,620	1,800-7,000	/μL
Eosinophil count	6.6	1-4	%
C-reactive protein (CRP)	0.21	<0.3	mg/dL
Serum amylase (AMY)	82	44-132	U/L
Creatine kinase (CK)	97	59-248	U/L
CK-MB	2	≤6	%
Aldolase	4.1	2.1-6.1	IU/L/37°C
Myoglobin	43.7	≤60	ng/mL
Anti-Jo-1 antibody	Negative	Negative	-

**Figure 7:**
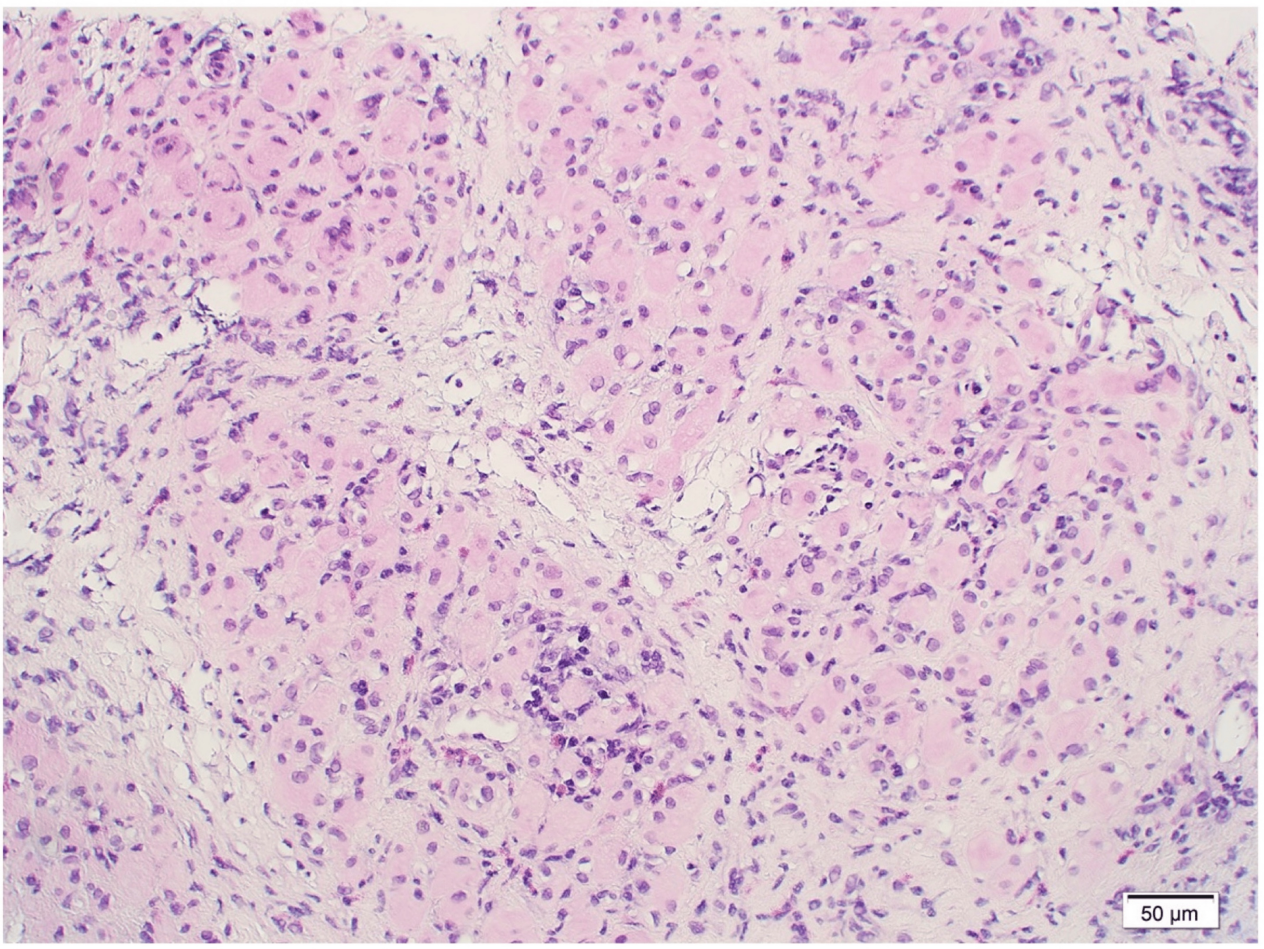
Histopathological findings of the right masseter muscle biopsy (200× magnification). Hematoxylin-eosin staining reveals moderate inflammatory cell infiltration, including lymphocytes, plasma cells, and eosinophils, accompanied by muscle atrophy.

Based on the imaging findings of bone sclerosis, suspected mandibular osteomyelitis was diagnosed, and empirical therapy with sulbactam sodium and ampicillin sodium was started at 12 g/day. A needle biopsy of the right masseter muscle was performed on day three, prompted by the elevated eosinophil count and the unusual presentation of masseter swelling with minimal inflammatory markers. Histopathological examination showed moderate inflammatory cell infiltration, including lymphocytes, plasma cells, and eosinophils, and muscle atrophy, confirming a diagnosis of FM (Figure 7).

There were no significant eosinophil clusters observed in the specimen. On day six, the antibiotic regimen was switched to oral amoxicillin and clavulanic acid; however, this regimen was discontinued due to suspicion of a drug-induced rash. Prednisolone therapy (20 mg/day) was initiated for focal myositis, and clarithromycin (400 mg/day) was continued for suspected osteomyelitis. On day seven, swelling and pain in the right mandibular region and masseter muscle had improved, but the trismus persisted, and the patient was instructed to continue mouth-opening exercises.

On day 25, treatment specifically targeting FM was optimized based on histopathological findings and clinical response. Anti-Jo-1 antibody test was negative (<1.0; reference range: <10.0), further supporting the diagnosis of FM rather than polymyositis. The patient continued on oral prednisolone therapy (20 mg/day), which was tapered to 15 mg/day after two weeks and 10 mg/day after four weeks, considering the patient's advanced age and HBV carrier status. Prior to initiating steroid therapy, the risks of HBV reactivation were explained to the patient, and informed consent was obtained. Throughout the treatment period, liver function tests remained stable, though HBsAg was not reassessed.

**Figure 8:**
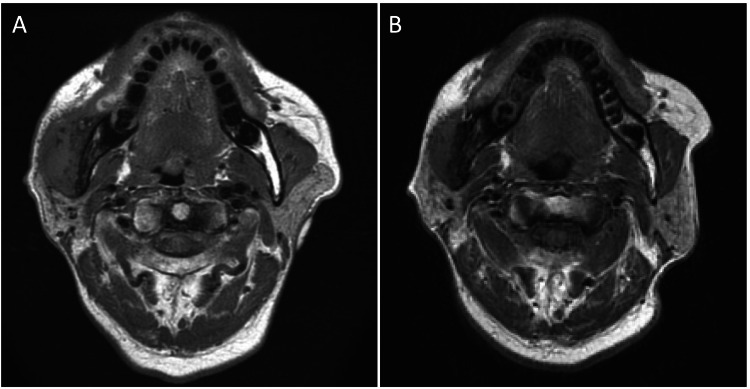
MRI findings of the masseter muscle before and after oral corticosteroid therapy. (A) Pre-treatment image showing significant swelling of the right masseter muscle (arrow). (B) Posttreatment image obtained one month after the initiation of oral corticosteroid therapy showing a marked reduction in muscle swelling (arrow).

By four weeks, MRI revealed a significant reduction in masseter muscle swelling, as shown in Figure 8A (pre-treatment) and Figure 8B (post-treatment).

This reduction correlated with improved clinical outcomes, including an increase in maximum mouth opening from 5 to 14 mm during treatment. The prednisolone dose was further tapered to 7.5 mg/day after two months and to 5 mg/day after three months. After five months, the prednisolone dose was reduced to 2.5 mg/day, and the symptoms were stable, with no recurrence of swelling or pain.

Regarding the right mandibular second molar, which may have been associated with the osteomyelitis, extraction was considered but deemed difficult due to the persistent trismus. Conservative management was therefore continued. The antibiotic therapy included five days of sulbactam sodium and ampicillin sodium, followed by clarithromycin for an extended period (56 days according to records).

Despite the improvement in inflammation, a persistent limitation of mouth opening (12 mm) was noted, likely due to fibrosis observed on initial imaging. Surgical intervention for persistent trismus was considered but was deferred due to concerns regarding scarring and recurrence. Conservative treatment with mouth-opening exercises continued.

## Discussion

The diagnosis of FM in the head and neck region is challenging because of its rarity and overlapping symptoms with more prevalent conditions, such as TMD, neoplasms, and infectious myositis [6]. In this case, the initial consideration of TMD was reasonable based on the patient's history and clinical presentation. However, retrospective analysis of the initial MRI revealed subtle edema-like changes in the masseter muscle, which might have represented early manifestations of FM. This highlights the importance of thoroughly evaluating all imaging findings, even when the primary diagnosis seems evident.

The persistence of trismus despite conservative TMD management should raise suspicion for alternative diagnoses. In our case, the worsening of symptoms after 15 months, characterized by increasing trismus and masseter swelling, prompted a reevaluation that led to the correct diagnosis. The question remains whether the patient had concurrent TMD and FM from the outset, or if the TMD-like symptoms were early manifestations of FM. The persistence of trismus even after successful treatment of FM inflammation suggests that either irreversible fibrotic changes had occurred in the masseter muscle due to chronic FM or that the patient indeed had concurrent TMD that remained unresolved.

The differential diagnosis between FM and pyogenic masseteritis or odontogenic infection was crucial in this case. Several clinical findings supported our diagnosis of FM over infectious etiology. First, blood tests showed no significant inflammatory markers (white blood cell count (WBC: 6,000/μL, C-reactive protein (CRP): 0.21 mg/dL) despite acute symptoms, which is atypical for bacterial infection. Second, the elevated eosinophil count (6.6%) suggested an inflammatory process rather than bacterial infection, although Auerbach et al. [8] reported that eosinophilic infiltration can be a feature of FM. In our case, histopathological examination showed eosinophils without significant clustering, supporting the diagnosis of FM rather than eosinophilic polymyositis. Third, the patient was afebrile (36.1°C), which is inconsistent with acute infectious myositis. Fourth, although there was evidence of mandibular osteomyelitis based on imaging findings, clinical examination did not reveal typical signs of odontogenic infection such as percussion pain or persistent purulent discharge from the right mandibular second molar. While there was a record of purulent discharge three months prior to acute presentation, no acute symptoms were present at the initial diagnosis of TMD when trismus was already evident. Finally, DWI showed high signal intensity in the masseter muscle, which is characteristic of inflammatory processes rather than purely infectious conditions. DWI has been reported to be particularly useful in identifying active inflammation in myositis, and our findings support its diagnostic utility [9]. The concurrent presence of bone sclerosis, suggesting chronic osteomyelitis, complicates the clinical picture. It is possible that chronic low-grade infection contributed to the local environment that predisposed to FM, or that they were separate, concurrent processes. The significant improvement in masseter swelling with corticosteroid therapy, despite persistent bone changes, supports the primary diagnosis of FM.

The etiology of FM remains largely unclear, with evidence suggesting a multifactorial origin. The potential mechanisms include nerve lesions, such as denervation-induced inflammation caused by radiculopathy or nerve trunk injury, and mechanical injury from trauma or intramuscular malformations. Infectious agents, including viruses, bacteria, fungi, and parasites, have also been thought to play a role, while immunological diseases, such as Behçet’s disease, systemic lupus erythematosus, and Sjögren’s syndrome, suggest an autoimmune component in certain cases. Iatrogenic factors, such as statin use, further expand the scope of potential triggers [2]. Despite these insights, many cases are idiopathic, and further research into genetic predisposition, environmental factors, and novel biomarkers such as myositis-specific autoantibodies and microRNAs is needed to improve our understanding of FM pathogenesis [7].

MRI depicts the characteristic findings of FM, such as muscle swelling, edema, and fibrosis and is extremely useful in distinguishing FM from malignancies and systemic myopathies [2]. However, histopathological confirmation is essential for definitive diagnosis. In this case, the findings of lymphocytic infiltration, muscle fiber necrosis, and interstitial fibrosis observed in the muscle biopsy were important in excluding malignant tumors and systemic inflammatory myopathy, and were consistent with previously reported features of FM [7]. The presence of concomitant osteomyelitis in our case further emphasized the importance of comprehensive diagnostic evaluation.

Corticosteroids are the primary treatment for FM because they can reduce inflammation and alleviate symptoms. In this case, prednisolone at 20 mg/day proved effective in reducing masseter swelling, as evidenced by follow-up MRI. However, in chronic or refractory cases, additional interventions such as immunosuppressive therapy or localized radiation may be necessary [10]. The treatment approach required careful consideration due to the patient's HBV carrier status and advanced age. The risk of HBV reactivation with corticosteroid therapy is well-documented [11], necessitating close monitoring and potentially antiviral prophylaxis. In our case, informed consent was obtained after explaining the risks, and liver function tests were monitored throughout treatment, though HBsAg levels were not reassessed. The tapering schedule (20 mg/day initially, reduced to 15 mg/day after two weeks, then 10 mg/day after four weeks, etc.) was designed to minimize both the risk of FM recurrence and potential complications from prolonged steroid use. This approach appears to have been successful, as the patient showed significant clinical improvement without recurrence or complications during the follow-up period. While the swelling and pain improved significantly with oral prednisolone, trismus persisted due to advanced fibrotic changes at the time of diagnosis. This experience highlights two important points: first, the importance of early intervention to prevent irreversible structural damage, and second, the need for a customized management strategy in patients with coexisting complications such as osteomyelitis, combining corticosteroids, antibiotic therapy, and physical rehabilitation. The management of the concurrent suspected osteomyelitis with antibiotics (initially sulbactam sodium and ampicillin sodium, followed by clarithromycin) demonstrates the importance of addressing all potential etiological factors. The long-term antibiotic therapy (56 days of clarithromycin) was likely extended due to concerns about potential infection exacerbation during immunosuppressive therapy.

This case emphasizes several important clinical lessons. Primarily, FM should be considered in the differential diagnosis of unilateral muscle swelling and trismus when conventional treatment fails. Additionally, comprehensive diagnostic evaluation, including MRI with DWI and muscle biopsy, is invaluable in cases of atypical or treatment-resistant masticatory muscle symptoms. Early intervention is crucial to prevent irreversible fibrotic changes that may lead to persistent functional limitations. Complex cases involving multiple potential etiologies benefit significantly from a multidisciplinary approach. Furthermore, DWI has proven particularly useful in identifying inflammatory muscle lesions, potentially enabling earlier diagnosis.

## Conclusions

Despite successful treatment of inflammation, our patient experienced persistent trismus, likely due to fibrotic changes that had already occurred before definitive diagnosis and treatment. This underscores the importance of timely diagnosis and intervention in FM cases to preserve function and prevent long-term sequelae.
